# Comparison of PRRSV Nucleic Acid and Antibody Detection in Pen-Based Oral Fluid and Individual Serum Samples in Three Different Age Categories of Post-Weaning Pigs from Endemically Infected Farms

**DOI:** 10.1371/journal.pone.0166300

**Published:** 2016-11-07

**Authors:** Nick De Regge, Brigitte Cay

**Affiliations:** Enzootic and (Re)emerging Diseases, Operational Direction Viral Diseases, CODA-CERVA, Groeselenberg 99, 1180 Ukkel, Belgium; Friedrich-Loeffler-Institut, GERMANY

## Abstract

**Background:**

Porcine reproductive and respiratory syndrome virus (PRRSV) is the causative agent of an economically important disease in swine. Since it has been shown that PRRSV and PRRSV specific antibodies can be detected in oral fluid, many different aspects have been studied to show that oral fluid could be a worthy alternative diagnostic sample to serum for monitoring and surveillance of this disease. Thorough field evaluations are however missing to convincingly show its usefulness under representative field conditions.

**Methodology:**

Pen-based oral fluid samples and serum samples from all individual pigs in the corresponding pens were collected from post-weaning pigs of three different age categories in eight endemically PRRSV infected farms and one PRRSV free farm in Belgium. All samples were tested by quantitative reverse transcription polymerase chain reaction (qRT-PCR) and ELISA to detect PRRSV RNA and PRRSV specific antibodies, respectively.

**Results:**

While the relative specificity of PRRSV detection by qRT-PCR in pen-based oral fluid compared to serum collected from individual pigs was high in all age categories (>90%), the relative sensitivity decreased with the age of the pigs (89, 93 and 10% in 8-12w, 16-20w and 24-28w old pigs, respectively). The latter correlated with a lower percentage of PRRSV positive pigs in serum/pen in the different age categories (55, 29 and 6%, respectively). Irrespective of the age category, pen-based oral fluid samples were always found PCR positive when at least 30% of the individual pigs were positive in serum. PRRSV specific antibody detection in oral fluid by ELISA showed a 100% relative sensitivity to detection in serum since oral fluid samples were always positive as soon as one pig in the pen was positive in serum. On the other hand, two false positive oral fluid samples in 11 pens without serum positive pigs were found, resulting in a relative specificity of 82%. Indications are however present that the oral fluid result indicated the correct infection status but the absence of a golden standard test makes it difficult to define definitive test characteristics.

**Conclusions:**

Overall it can be concluded that oral fluid seems to be a useful matrix for diagnosis of PRRSV under field conditions and that differences in kinetics of PRRSV and PRRSV specific antibody detection in oral fluid and serum of individual pigs can also be reflected in pen-based oral fluid results.

## Introduction

*Porcine reproductive and respiratory syndrome virus* (PRRSV) is the etiologic agent of PRRS, a chronic, infectious and economically important disease of swine that is still difficult to control despite the availability of different types of vaccines [1]. PRRSV is differentiated into genetically distinct genotype 1 (European genotype) and genotype 2 (American genotype) strains. In Western and Central Europe, including Belgium, mostly genotype 1 (subtype 1) strains are circulating [2]. Recently the presence of a non-vaccine related genotype 2 strain was reported in Hungary [3].

For a long time, serum collected from individual pigs has been considered as the best diagnostic sample for monitoring and surveillance of this disease. Since it has been repeatedly reported that PRRSV and PRRSV specific antibodies can also be detected in porcine oral fluid [4–8], efforts have been undertaken to evaluate whether this could represent an alternative diagnostic matrix to the routinely used serum. Oral fluid collected at pen level namely possesses several benefits compared to blood sampling: it is a non-invasive collection technique; samples can be collected at group level, and time and money can be saved due to the ease of collection. Many different aspects related to oral fluid sampling and its use as a diagnostic matrix for PRRSV detection have been studied during the past decade: RNA extraction methods and PCR tests were compared and developed for PRRSV detection [1,9–10]; tests for PRRSV specific antibody detection of different isotypes were developed and compared [11–14]; issues related to collection material, sample collection protocol and sample storage were studied and optimized [10,14–17]; and comparison was made between oral fluid and serum diagnostics for PRRSV in individual pigs [18–23]. All these studies showed that oral fluid could be a promising matrix for PRRSV surveillance.

The last and crucial step in the evaluation process is to compare diagnostic results obtained in pen-based oral fluid to results in serum from the corresponding individual pigs in that pen. This has only been studied under experimental conditions [24] and in a small scale field study [25]. Therefore the objective of this study was to perform this comparison on a larger scale and in a setting representative for European pig farming conditions. PRRSV and PRRSV specific antibody detection were compared in pen-based oral fluid and serum samples collected from pigs of three different age categories in eight Belgian farms endemically infected with PRRSV.

## Materials and methods

### Ethics statement

The pig farmers participating in this study gave permission to conduct the study on their premises. Approval for this study was granted by the joined ethical committee of CODA-CERVA and the Institute of Public Health Belgium, but no specific dossier had to be filed since the collection of blood and oral fluid from pigs at the farm by a veterinarian is considered as a routine veterinary practice and needs no specific approval from an ethical committee under current European and Belgian legislation (Directive 2010/63/EU of the European parliament and of the council of 22 September 2010 on the protection of animals used for scientific purposes; Belgian Royal Decree of May 2013 relating to the accommodation and care of experimental animals (C 2013/24221, chap I. §4)). The samples were collected for the purpose of this study between December 2012 and January 2014 and the results were communicated to the farmers.

### Study population and sample collection

A field study was carried out in nine single site farrow-to-finish herds with a four-week batch management production system located in Flanders (Belgium) (F1-F9; Table 1). Eight herds were known to be endemically infected by PRRSV and were included on the basis of known seropositive PRRSV results during the year before sampling (F1-F8). None of them vaccinated the piglets while seven out of eight herds vaccinated pregnant sows and guilts with a live attenuated vaccine or a combination of a live attenuated and an inactivated vaccine. One herd (F9) was selected as a negative control herd since it had no history of PRRSV and is screened regularly to maintain its negative status.

**Table 1:** Summary of infection and vaccination status of farms where pen-based oral fluid and serum samples were collected.

farm	province	PRRSV status	vaccination	visits	pens	sampled pigs	PCR	ELISA
F1	Antwerp	positive	no vaccination	3	9	107	x	x
F2	West Flanders	positive	yes, but no details	3	9	107	x	x
F3	West Flanders	positive	inactivated + life attenuated	3	9	137	x	x
F4	Antwerp	positive	inactivated + life attenuated (1x)	3	9	140	x	x
F5	Flemish Brabant	positive	yes, but no details	3	9	90	x	x
F6	Antwerp	positive	life attenuated	3	9	91	x	
F7	Antwerp	positive	life attenuated	3	9	92	x	
F8	Antwerp	positive	yes, but no details	1	3	49		x
F9	Hainaut	negative	no vaccination	1	7	59	x	x

Most herds (F1-F7) were visited 3 times with a 2 week interval. F8 and F9 were only visited once. In all herds and on each visit, three litters were selected: one in the post-weaning section, one at the beginning of the finishing phase and one at the end of the finishing phase. This corresponded with an approximate age of the pigs of 8, 16 and 24 weeks at the first sampling visit. During the 2 subsequent visits, again one representative litter per section was selected. Only on F5, each time the same litters were sampled. At each visit, one pen-based oral fluid sample was collected from each litter by hanging a cotton rope in the pen, following the method described by Pricket et al. (2008) [5]. In brief, one rope (length 1m; diameter 14 mm) was suspended in the pen and left in place for 30 min, during which the pigs could chew on it and moisten it with oral fluid. The ropes were thereafter manually wrung to collect the oral fluid in a 50 mL conical centrifuge tube. Oral fluid samples were immediately chilled on ice. Upon arrival in the lab, samples were centrifuged at 1800 x g for 10 minutes at 4°C and stored as aliquots at -80°C until use. Besides the collection of oral fluid, all individual pigs from the litters were blood sampled to obtain serum. Pigs were restrained and drawn blood from the external jugular vein by venipuncture. Blood samples were allowed to clot for 1 h at room temperature and serum was harvested by centrifugation for 10 min at 1800 x g and stored at −80°C until further testing. Oral fluid sampling was conducted before blood sampling to prevent that animals would refuse to bite on the ropes after physical manipulation and all sampling was carried out by the same personnel in order to reduce bias. In total 872 pigs belonging to 73 pens were sampled and each pen contained a minimum of 7 and a maximum of 30 pigs.

### Detection of porcine reproductive and respiratory syndrome virus by quantitative reverse transcription real-time PCR

PRRSV RNA in serum and oral fluid was detected via a qRT-PCR targeting an ORF 7 fragment (TaqMan NA and EU PRRSV Reagents, Thermofisher Scientific, Gent, Belgium). Briefly, total RNA from serum samples was extracted using the MagMAX Pathogen RNA/DNA kit (Thermofisher Scientific, Gent, Belgium) according to the manufacturer’s protocol. RNA from oral fluid was extracted as previously described [10]. Briefly, 150μl oral fluid was mixed with 225 μl of lysis/binding solution and 1μl carrier RNA, followed by 3 minutes of vortexing. After a centrifugation step of 2 min at 13000 tpm, 115 μl of supernatans was added to 20 μl bead mix and 65 μl isopropanol. Further washing and extraction were done following the automated protocol of the manufacturer (MagMAX Pathogen RNA/DNA kit, Thermofisher Scientific, Gent, Belgium). After the RNA extraction, commercial TaqMan NA and EU PRRSV reagents (Thermofisher Scientific, Gent, Belgium) were used for PRRSV detection by PCR. 25 μl reaction mixtures containing 12.5 μl 2x Multiplex RT-PCR Buffer, 2.5 μl 10x PRRSV Primer Probe mix, 1.25 μl Multiplex Enzyme Mix, 0.75 μL Nuclease-free Water and 8 μl of extracted RNA were prepared and run on a LightCycler 480 Real-time PCR system (Roche, Switzerland) following the temperature cycle prescribed by the manufacturer. For each assay, positive and negative control samples were tested with the unknowns. Samples with a Ct < 37 were considered positive. For comparison of results in oral fluid and serum, a litter was considered PRRSV positive as soon as one serum sample originating from that litter tested positive.

### Detection of PRRSV-specific antibodies by enzyme-linked immunosorbent assays

Commercially available HerdChek® PRRS 3X ELISA and PRRS OF ELISA (IDEXX Laboratories Inc., Westbrook, ME ELISA) were used to evaluate the presence of PRRSV-specific Ig G antibodies in serum and oral fluid samples, respectively. The manufacturer quotes sensitivities of 98.8 and 98% and specificities of 99.9 and 98% for the serum and oral fluid ELISAs, respectively. Both assays were performed according to the manufacturer’s instructions. ELISA results were expressed as S/P ratios (OD value of sample–OD value of negative control) / (OD value of positive control–OD value of negative control). Samples with an S/P value equal or greater than the cut-off value (0.40) were considered positive for PRRSV-specific antibodies. For comparison of results in oral fluid and serum, a litter was considered positive for PRRSV specific antibodies as soon as one serum sample originating from that litter was found positive in ELISA.

### Statistical analysis

A one-way ANOVA analysis and Tukey post hoc tests were used to evaluate whether there were significant differences in the percentage of PRRSV PCR positive pigs in serum per pen between different age groups.

The agreement between the detection of PRRSV by PCR in serum and saliva, respectively, and of PRRSV specific antibodies by ELISA in serum and saliva, respectively, was measured using Cohen’s kappa coefficient. The following ranges were used for interpretation of the kappa coefficient [26]: poor agreement: 0.00; slight agreement: 0.00–0.20; fair agreement: 0.21–0.40; moderate agreement: 0.41–0.60; substantial agreement: 0.61–0.80; almost perfect: 0.81–1.00.

A linear regression was performed to analyze whether S/P values obtained in serum can be used to predict S/P values in pen-based oral fluid samples.

Data were analyzed using SPSS Statistics V22.0 software and P values < 0.05 were considered to be significant.

## Results

### Detection of porcine reproductive and respiratory syndrome virus by quantitative reverse transcription real-time PCR

**Fig 1:**
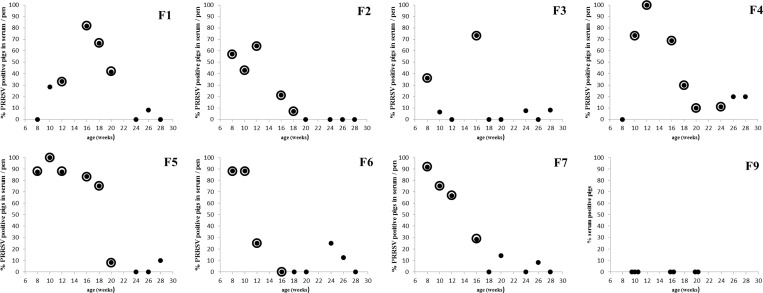
Overview of PRRSV detection by qRT-PCR in serum and oral fluid collected on seven endemically PRRSV infected farms (F1-F7) and one PRRSV free farm (F9). Full dots indicate the percentage of PRRSV positive pigs in serum per pen. If the full dot is surrounded by an open circle, it indicates that the corresponding pen based oral fluid sample was positive in qRT-PCR.

A total of 823 pigs belonging to 70 pens were tested in qRT-PCR. An overview of the PCR results in serum and oral fluid per farm can be found in Fig 1. Table 2A summarizes the concordance between both matrices in a cross table. While 57 out of 70 pens obtained the same PRRSV infection status in oral fluid and in serum, 12 pens were found PRRSV negative in oral fluid while at least one animal/pen was found positive in serum (F1: 10w + 26w; F3: 10w + 24w + 28w; F4: 26w + 28w; F5: 28w; F6: 24w + 26w; F7: 20w + 26w). On the other hand, one pen was found positive in oral fluid while all serum samples from the corresponding pen were negative (F6: 16w). This resulted in a relative sensitivity, relative specificity and relative trueness of respectively 71%, 96% and 81% of PRRSV detection by PCR in pen-based oral fluid compared to individual serum samples, and a kappa value of 0,637.

**Table 2:** Comparison of PRRSV detection in oral fluid and serum via qRT-PCR (a) and overview of relative test characteristics and mean percentage of PRRSV positive pigs per pen for the different age categories (b). A litter was considered PRRSV positive in serum as soon as one serum sample originating from that litter tested positive.

**a.**		** **	**OF**						
	** **	** **	**pos**	**neg**	** **				
	**serum**	**pos**	30	12	42				
		**neg**	1	27	28				
		** **	31	39	70				
**b.**	** **	**relative sensitivity**		**relative specificity**		**kappa**	**mean (± SEM) % PRRSV positive pigs / pen**		
	overall	71		96		0,637	30 ± 4		
	8–12	89		100		0,8	55 ± 8		
	16–20	93		91		0,838	29 ± 7		
	24–28	10		100		0,104	6 ± 2		

**Fig 2:**
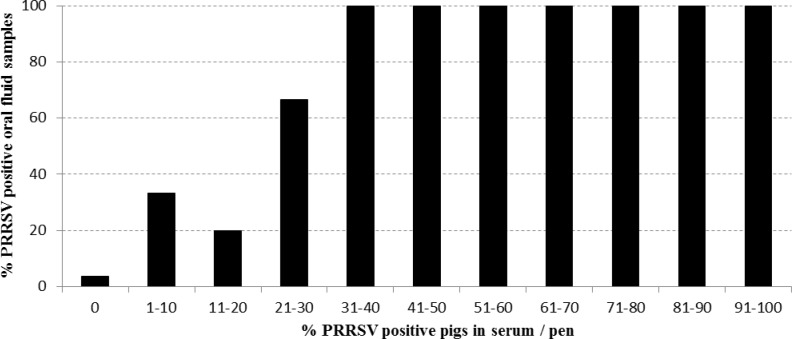
Probability to detect PRRSV in a pen based oral fluid sample by qRT-PCR dependent on the percentage of PRRSV positive pigs in serum per pen.

It was clear that most false negative results (9/12) were found in the oldest age category (24–28 weeks). Interestingly, this is in line with the observation that the mean percentage of PRRSV PCR positive pigs in serum/pen of 24–28 week old pigs (6%) is significantly lower (ANOVA: p < 0,001; all Tukey post hoc tests: p < 0,05) than in 8-12w (55%) and 16-20w (29%) old pigs (Table 2B). Therefore, when the relative sensitivity between test results in oral fluid compared to serum is determined per age category, a relative sensitivity of about 90% is found in pigs of 8-12w and 16-20w, while this is only 10% in 24-28w old pigs (Table 2B). This indicates that a minimum percentage of pigs per pen needs to be PCR positive in serum to allow a reliable detection in oral fluid. Our results show that when, irrespective of the pigs age, at least 30% of the individual pigs/pen were PRRSV positive in serum, also the corresponding pen-based oral fluid sample was always PCR positive (Fig 2). The relative specificity of PCR testing in oral fluid compared to serum was high (> 90%) independent of the age category (Table 2B).

### Detection of antibodies directed against porcine reproductive and respiratory syndrome virus by ELISA

**Fig 3:**
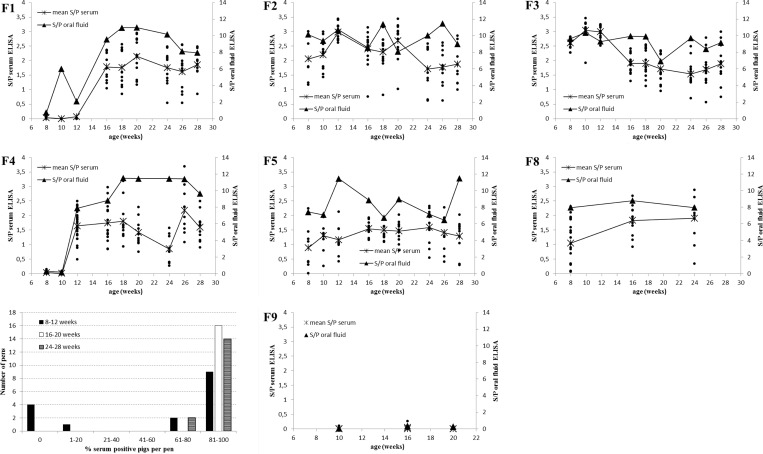
Overview of PRRSV specific antibody detection by ELISA in serum and oral fluid collected on six endemically PRRSV infected farms (F1-F5; F8) and one PRRSV free farm (F9). The S/P value obtained for oral fluid is indicated by a triangle and the mean S/P value of sera from all pigs in a pen is indicated by a cross. Individual S/P values in sera from all pigs in a pen are shown in full dots. The histogram summarizes the PRRSV seroprevalence in the three age categories of pigs from the endemically PRRSV infected farms (F1-F5; F8).

A total of 689 pigs belonging to 55 pens were tested in ELISA and all results are summarized in Fig 3. Among the farms with a known PRRSV history, a high seroprevalence (60–100% of pigs/pen) of PRRSV specific antibodies was found. Only in 4 out of 48 pens (F1: 8w + 10w; F4: 8w + 10w), all pigs were negative. All these animals belonged to the youngest age category (8–12 weeks) and originated from the non-vaccinated herd (F1) or from the herd where guilts and sows were vaccinated only once (F4). The negative status of F9 was confirmed by absence of PRRSV specific antibodies.

**Fig 4:**
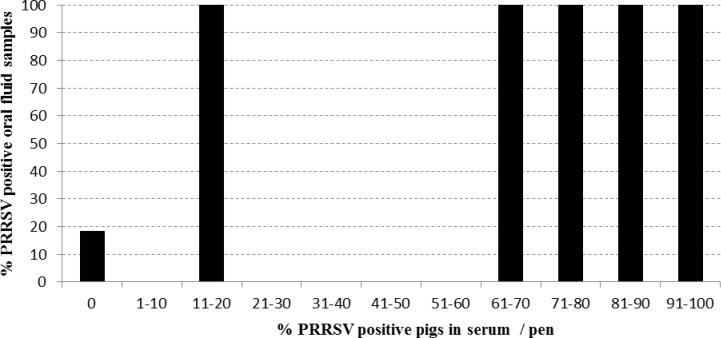
Probability to detect PRRSV specific antibodies in a pen based oral fluid sample by ELISA dependent on the percentage of PRRSV antibody positive pigs in serum per pen.

Table 3 summarizes the concordance between both matrices in a cross table. A high relative trueness of 96% was found since 53 out of 55 pens obtained the same PRRSV status in ELISAs on pen-based oral fluid and individual serum samples. A relative sensitivity of 100% was found since all pens containing at least one pig that was ELISA positive in serum (Fig 4) were also found positive in the corresponding oral fluid sample. Two pens (F1: 8w + 10w) scored positive in oral fluid while all individual animals tested negative in serum, resulting in a relative specificity of 82%. Interestingly, the S/P value in the oral fluid sample of F1: 8w was only just above the cut-off (0,74) and 2 out of 7 pigs in the pen F1: 10w were PCR positive (Fig 1).

**Table 3:** Comparison of PRRSV specific antibody detection in oral fluid and serum via ELISA. A litter was considered PRRSV positive in serum as soon as one serum sample originating from that litter tested positive.

		OF		
		pos	neg	
**serum**	**pos**	44	0	44
	**neg**	2	9	11
	** **	46	9	55

**Fig 5:**
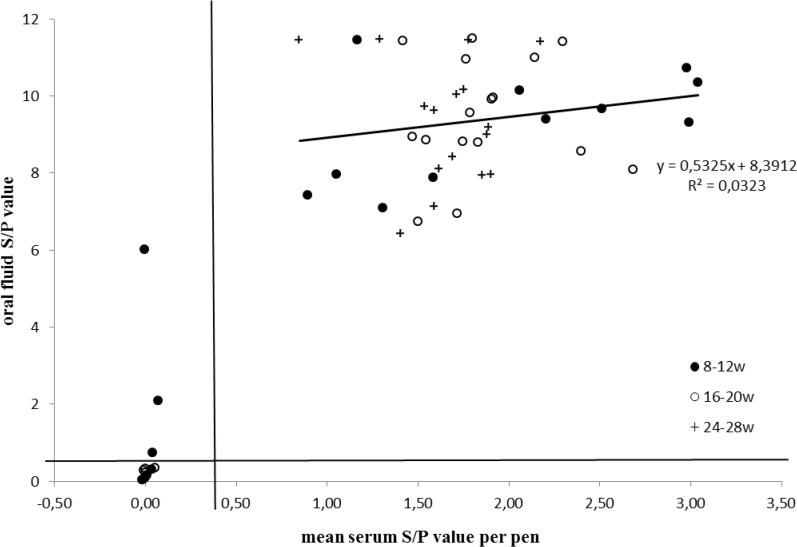
Linear regression analysis of S/P values of pen-based oral fluid and mean S/P values of individual serum samples in the corresponding pen.

Fig 5 shows the correlation between the mean S/P value obtained in serum per pen and the S/P value obtained for the corresponding pen-based oral fluid sample. When only the data with positive S/P values (> 0,4) are considered, a linear regression analysis showed that the S/P values obtained in serum cannot be used to predict the S/P value in oral fluid (P = 0,246).

## Discussion

In order to convincingly show that collection of pen-based oral fluid can be a valid alternative to the routinely used blood sampling for diagnosis and surveillance of PRRSV, it is important to compare diagnostic test results in both sample types collected under representative field conditions. In this study, samples were therefore collected from pigs of three different age categories in eight farms endemically infected with PRRSV. The PRRSV infection status was confirmed by the detection of circulating virus in post-weaning pigs and high prevalence of PRRSV specific antibodies in finishing pigs, corresponding with earlier reported patterns of virus circulation and antibody development on endemically infected farms [27–28].

Firstly, we compared PRRSV RNA detection in both matrices via qRT-PCR. Our results showed that the chance to detect PRRSV in a pen-based oral fluid sample correlated with the percentage of PCR positive pigs in serum per pen. Irrespective of the age category, pen-based oral fluid samples were always found PCR positive when at least 30% of the individual pigs where positive in serum. This is line with the results of Olsen et al. (2013) [24] who reported a probability of more than 90% to detect PRRSV by PCR in pen-based oral fluid when a pen contained 36% of vaccinated pigs. Since the mean number of PCR positive pigs per pen decreased over time, the relative sensitivity of PRRSV detection in oral fluid compared to serum showed to decrease in the different age categories that were sampled. While it was high (90%) in pens containing pigs of 8-12w and 16-20w, it was only 10% in pens containing late finishing pigs of 24-28w. The most plausible explanation for the latter can probably be found in the observation that only 6% of the individual pigs/pen were positive in serum in this oldest age category, what in practice mostly corresponded to one animal per pen harboring low virus load (data not shown). While previous studies have shown that about 80% of pigs chew on a rope within 30 min after introduction in the pen [29], it is possible that the positive animal did not chew on the rope. Alternatively, the low virus load could have been further diluted in the pool of saliva of the other chewing pigs and have dropped below the detection limit. A reduced sensitivity of qRT-PCR testing on oral fluid from individual pigs that were only weak positive in serum has already been documented before [20]. The same reasoning could hold true for the two pens that were missed in the youngest age category (8-12w). The negative oral fluid result in the non-vaccinating farm (F1: 10w) at the moment that virus circulation was detected for the first time in serum could however potentially also be explained by our previous finding that oral fluid becomes later PRRSV positive than serum after initial infection [1]. Before concluding that PRRSV monitoring in oral fluid is less sensitive than monitoring in serum, it should also be noted that current monitoring and surveillance programs that are based on blood sampling usually not sample all animals in the pen but often only select and test 5 animals per age category. As a result, pens containing only one or two PRRSV positive pigs could also be missed by that type of monitoring system.

Furthermore, qRT-PCR on oral fluid showed an overall high relative specificity (96%) compared to serum testing. Only one pen out of 28 (F6: 16w) containing pigs that were all negative in serum was found positive in oral fluid. Interestingly for this particular case, high PRRSV presence that started to decrease had been found in the 8-12w age category. The positive oral fluid sample at 16w could therefore be a field example of the fact that oral fluid remains longer PCR positive than serum after PRRSV infection. This was described earlier during a comparison of serum and oral fluid diagnostics in individual pigs [19] and would imply that oral fluid could sometimes allow a more sensitive PRRSV detection than serum sampling. The observed high specificity of oral fluid sampling also indicates that it could be an appropriate tool to monitor the negative status of PRRSV free farms.

Several studies in literature have already reported good agreement between ELISA results in serum and oral fluid, both in samples from individual animals and in pen-based samples [19–20;25]. Also in our current study, the oral fluid ELISA showed to be highly sensitive. As soon as one animal in a pen was found PRRSV antibody positive in serum, the corresponding pen-based oral fluid sample was found positive. In contrast to the 100% sensitivity, a somewhat lower relative specificity of 82% (2/11) was found. Although this indicates that potentially false positive results could be obtained, it should be carefully considered that only 11 pens with all seronegative animals were present in this study and that indications are present that the obtained positive result in at least one of both positive samples could reflect the correct infection status. Two out of 7 animals in the pen containing 10w old pigs from the non-vaccinating farm (F1) were namely PCR positive. Unfortunately, there is no golden standard test available to irrefutably determine the correct infection status. The existence of false positive results in oral fluid have however already been reported before in samples collected from individual animals [18] and thus deserves further attention.

In practice, several diagnostic labs tend to use the obtained S/P values with the HerdChek® PRRS 3X ELISA (IDEXX) in serum to estimate whether positive ELISA results indicate recent infections or antibodies induced by vaccination (personal communication). The fact that we did not find a significant linear regression between the S/P values of pen-based OF samples and the corresponding mean S/P values in serum per pen for pens containing PRRSV antibody positive pigs indicate that S/P values in serum cannot be used to predict pen-based S/P values in oral fluid and indirectly that S/P values obtained with the PRRS OF ELISA (IDEXX) should only be used for qualitative data assessment.

## Conclusion

Comparison of PRRSV and PRRSV specific antibody detection by PCR and ELISA, respectively, in pen-based oral fluid and serum samples collected from post-weaning pigs of different age categories showed that oral fluid can be considered as a useful matrix for diagnosis of PRRSV under field conditions. On the one hand, PRRSV detection by qRT-PCR in oral fluid was highly specific and pen-based oral fluid samples were always qRT-PCR positive when at least 30% of the individual pigs were positive in serum. False negative results in pen-based oral fluid were found in pens containing pigs of the oldest age category (24–28 w) since these mostly contained only a low percentage of PCR positive pigs in serum. On the other hand, PRRSV specific antibody detection in oral fluid by ELISA showed a100% relative sensitivity since oral fluid samples were always positive as soon as one pig in the pen was positive in serum. The relative specificity in oral fluid was however lower (82%) and should be further investigated in future experiments.
